# Anti-tumor efficacy of oncolytic reovirus against gastrointestinal stromal tumor cells

**DOI:** 10.18632/oncotarget.23361

**Published:** 2017-12-18

**Authors:** Yusuke Inagaki, Eiji Kubota, Yoshinori Mori, Mineyoshi Aoyama, Hiromi Kataoka, Randal N. Johnston, Takashi Joh

**Affiliations:** ^1^ Department of Gastroenterology and Metabolism, Nagoya City University Graduate School of Medical Sciences, Mizuho-Ku, Nagoya, Japan; ^2^ Department of Gastroenterology, Nagoya City West Medical Center, Kita-Ku, Nagoya, Japan; ^3^ Department of Pathobiology, Nagoya City University Graduate School of Pharmaceutical Sciences, Mizuho-Ku, Nagoya, Japan; ^4^ Department of Biochemistry and Molecular Biology, University of Calgary, Calgary, Alberta, Canada

**Keywords:** reovirus, gastrointestinal stromal tumor (GIST), imatinib, fas, apoptosis

## Abstract

Imatinib, a multitargeted receptor tyrosine kinase inhibitor, is used as the standard initial therapy against inoperable gastrointestinal stromal tumor (GIST). However, GIST can acquire resistance to imatinib within several years of therapy. The development of oncolytic reovirus as an anticancer agent has expanded to many clinical trials for various tumors. Here, we investigated whether reovirus has antitumor activity against GIST cells in the setting of imatinib sensitivity *in vitro* and *in vivo*. Cell proliferation and apoptosis assays were performed using a human GIST cell line, GIST-T1, and imatinib-resistant GIST (GIST-IR) cells that we established. The molecular pathways responsible for cell damage by reovirus were explored using PCR-arrays and Western blots. Reovirus significantly induced apoptotic cell death in GIST-T1 and GIST-IR cells *in vitro*, despite differences in the activation of receptor tyrosine kinase pathways between GIST-T1 and GIST-IR. Molecular assays indicated the possibility that reovirus induces apoptotic cell death via Fas signaling. Furthermore, *in vivo* mouse tumor xenograft models demonstrated a significant anti-tumor effect of reovirus on both GIST-T1 and GIST-IR cells. Our results demonstrate the therapeutic potential of reovirus against GIST.

## INTRODUCTION

Gastrointestinal stromal tumor (GIST) is the most common mesenchymal neoplasm of the digestive tract. GISTs are believed to originate from interstitial cells of Cajal (pacemaker cells of the digestive tract) or related stem cells, and are characterized by proto-oncogene receptor tyrosine kinase KIT or platelet-derived growth factor receptor alpha (PDGFRA) activating mutations [1, 2]. Activating mutations of KIT and PDGFRA genes permit ligand-independent phosphorylation of the receptor tyrosine kinase (RTK), perpetuating receptor-initiated signals and causing activation of downstream effectors. Increases in cellular proliferation and decreases in apoptosis lead to the development of neoplasia and enhance cell survival [2]. Imatinib mesylate, a tyrosine kinase inhibitor of KIT and PDGFRA receptors, is the first-line standard therapy for inoperable, metastatic, or recurrent GIST [2]. Although imatinib has been shown to improve survival in patients with GIST, drug resistance in GIST limits the effectiveness of imatinib. Approximately 5% of patients show primary resistance to imatinib and approximately 14% of patients develop early resistance; moreover, secondary resistance develops after a median of about 2 years of treatment with the drug [3, 4]. Resistance against imatinib can develop through various mechanisms, and mainly occurs due to additional kinase domain mutations [2, 5]. Sunitinib malate, a multitargeted receptor tyrosine kinase inhibitor, is used as a second-line standard therapy for imatinib-resistant GIST. Although sunitinib has clinical benefits including disease control and superior survival for imatinib-resistant GIST patients, it is ineffective for a cohort of imatinib-resistant GIST [4, 6]. Regorafenib, another multitargeted inhibitor, was recently approved as a third-line therapy, but shows limited efficacy [7]. Furthermore, sunitinib and regorafenib have higher rates of adverse events including hand-foot syndrome, diarrhea and hypertension [4, 7]. Therefore, it is necessary to elucidate new therapies and approaches for imatinib-resistant GIST.

Human oncolytic reovirus is a non-enveloped double-stranded RNA virus composed of an outer and inner protein shell, which altogether forms a 20-sided icosahedral capsid [8, 9]. Reovirus preferentially infects and kills transformed cancerous cells rather than healthy normal cells [10]. Though activation of the oncogenic Ras signaling pathway enhances reoviral oncolytic targeting in various types of human cancers, the mechanism of oncolysis by reovirus remains largely unknown [9–12]. The ability of reovirus to infect and lyse tumor cells under *in vitro* and *in vivo* conditions has been reported previously [13–16], and clinical trials involving various cancers are underway in the US, the UK, Belgium, and Canada. Several phase I/II clinical trials have demonstrated the safety of reovirus and its successful decrease of tumor size, leading to several phase II/III trials that are now ongoing [9, 16–18]. Despite many successes in translational studies and clinical trials of reovirus therapy against cancers, the efficacy of reovirus against GIST has not been reported to date.

In the current study, we investigated the antitumor activity of reovirus against GIST and imatinib-resistant GIST (GIST-IR), via modulating cell proliferation and apoptosis signaling *in vitro*, and whether mouse tumor xenograft models could demonstrate the anti-tumor effect of reovirus on GIST cells *in vivo*.

## RESULTS

### Characteristics of GIST-IR cells, imatinib-resistant GIST cells

**Figure 1:**
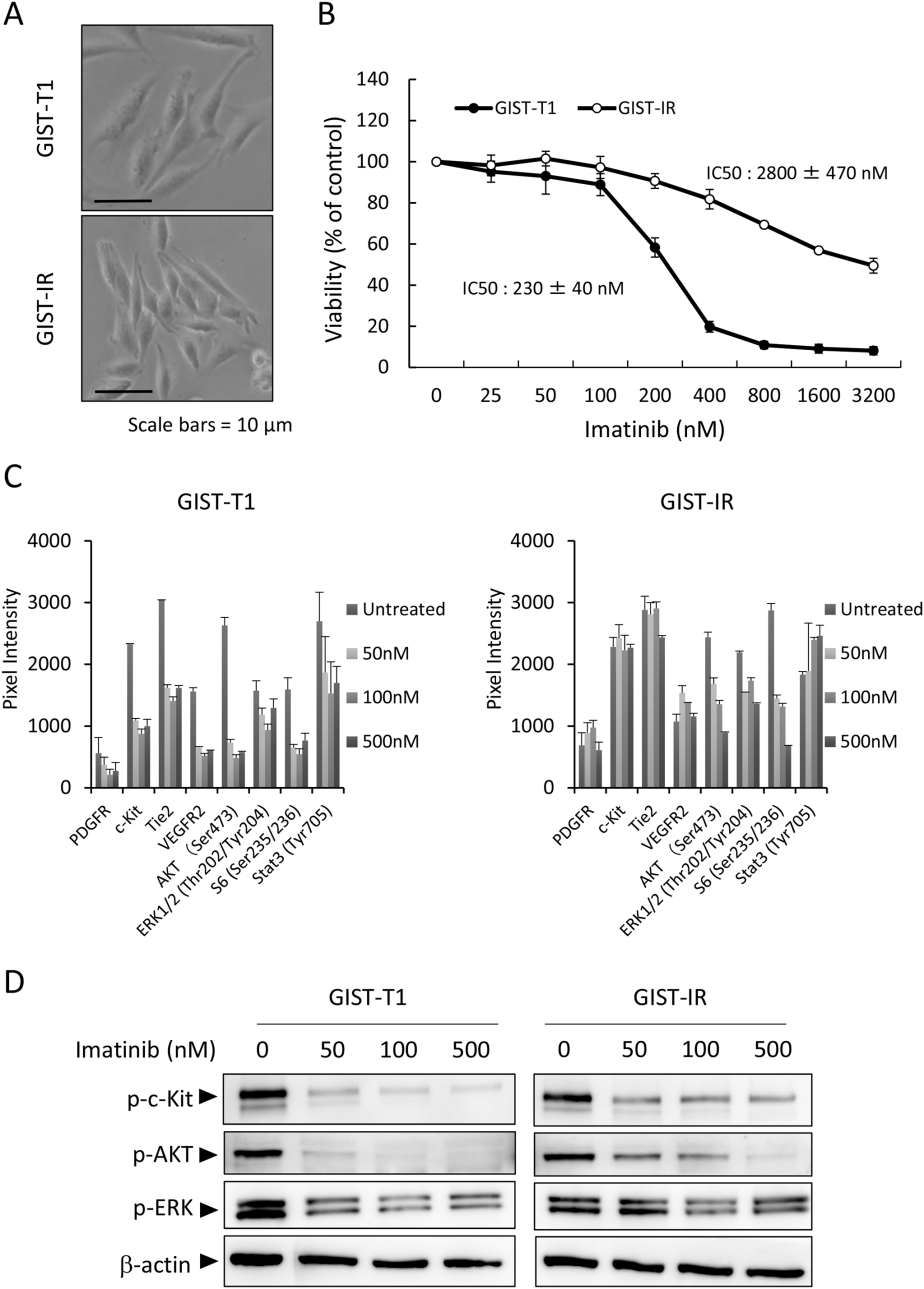
Characterization of the imatinib-resistant GIST cell line, GIST-IR **(A)** The appearance of imatinib-resistant GIST cells, GIST-IR cells, shows a spindle-like shape without obvious morphological changes from the parental cell, GIST-T1 cells. **(B)** 5.0 × 10^3^ GIST-T1 or GIST-IR cells were plated in 96-well plates and cultured with imatinib (25 - 3200 nM). After 48 hours, cell viability was measured by WST-8 assay. Imatinib showed efficacy against GIST-T1 and GIST-IR cells with IC50s of 230 ± 40 nM and 2800 ± 470 nM, respectively. **(C)** Expression of activated tyrosine kinase receptors and downstream signals in GIST-T1 and GIST-IR cells, and the effect of imatinib on their phosphorylation status were evaluated using RTK signaling antibody array. Bar graphs represent the quantified receptor tyrosine kinase (RTK) signaling antibody array images. Results represent the mean ± SD of three experiments performed in triplicate. **(D)** Protein extract from GIST-T1 and GIST-IR cells incubated in culture media supplemented with the indicated concentration of imatinib for 48 hours was immunoblotted with anti-c-Kit (Tyr719), anti-phospho-AKT (Ser473), anti-phospho-ERK1/2 (Thr202/Tyr204). β-actin is shown as a loading control.

We initially characterized the parental cell line, GIST-T1, and the imatinib-resistant cell line, GIST-IR. The GIST-IR cells appear spindle-shaped, very similar to the parental cell line, GIST-T1 (Figure 1A). The proliferation of GIST-IR cells was slower than that of GIST-T1 cells (Supplementary Figure 2). To assess the level of GIST-IR cellular resistance to imatinib, we determined the 50% growth inhibitory concentrations (IC50) for GIST-T1 and GIST-IR cells. Figure 1B shows that the IC50 of imatinib against GIST-T1 was 230 ± 40 nM and the IC50 of imatinib against GIST-IR was 2800 ± 470 nM, approximately twelve times higher than for the parental cell line. Secondary resistance to imatinib often develops due to secondary *KIT* or *PDGFRA* mutations that interfere with drug binding [2, 11, 19–21]. Most secondary *KIT* mutations represent single nucleotide substitutions affecting codons in exons 13, 14, 17 and 18 [2, 11, 19–21]. We performed targeted genome sequencing to examine whether GIST-IR cells have mutations that could contribute to drug resistance (Table 1), and did not find any of these gene mutations (data not shown). To analyze the resistant mechanism of GIST-IR cells against imatinib, we investigated differences in protein kinase activations between GIST-IR cells before and after treatment with imatinib, using a RTK phosphorylation array (Supplementary Figure 1). Results for the quantification of RTK phosphorylation array images are shown in Figure 1C. GIST-T1 cells phosphorylated c-Kit, Tie2, VEGFR2, AKT and ERK1/2, and their phosphorylation was inhibited in an imatinib concentration-dependent manner. In GIST-IR cells, several tyrosine kinase receptors and their downstream targets, including c-Kit, VEGFR2, Tie2, AKT, and ERK1/2 were strongly phosphorylated when compared with GIST-T1. In addition, the phosphorylation of these protein kinases did not decrease even in GIST-IR cells exposed to high imatinib concentrations. To ascertain the results of RTK phosphorylation array, we performed western blot and found that imatinib inhibited the phosphorylation of c-Kit, AKT, and ERK1/2 in GIST-T1 in a concentration dependent manner. In contrast, the phosphorylation of these protein kinases was not inhibited sufficiently in GIST-IR even though cells were exposed to high concentration of imatinib (Figure 1D).

**Table 1:** PCR primer sets for the analysis of KIT and PDGFRA mutations contributing to secondary imatinib resistance

Target	Direction	Primer
KIT 13	Forward	5′-ACCACCAGCACCATCACCACTTACCTTGTTGTCTT
	Reverse	5′-TTCTGCAAACATCACAGACATCCTTGATGGGAACT
KIT 14	Forward	5′-TTGGGACTAAGTAGTCTGATCCACTGAAGCTGAA
	Reverse	5′-TATACAGGAATAATCCAGAGGTCGATGGCAAGAA
KIT 17	Forward	5′-TGCAAAGGCATATTAGGAACTCTGTGAAAGGA
	Reverse	5′-AAATTTCTCCTGCTGTGACCTTCAATGACCTA
KIT 18	Forward	5′-GTTATCACTCCACATTTCAGCAACAGCAGCATCTA
	Reverse	5′-TCATTATGTCATACCTGCAAGACAAGGGCCATTTA
PDGFRA 18	Forward	5′-TCTTTGGGCATGCCTCTGCAACCTGATGATTTC
	Reverse	5′-GTGGAGCTGGCACTCAAACCTGGGCAATCTG

### Cytotoxic effect of reovirus against GIST cells *in vitro*

**Figure 2:**
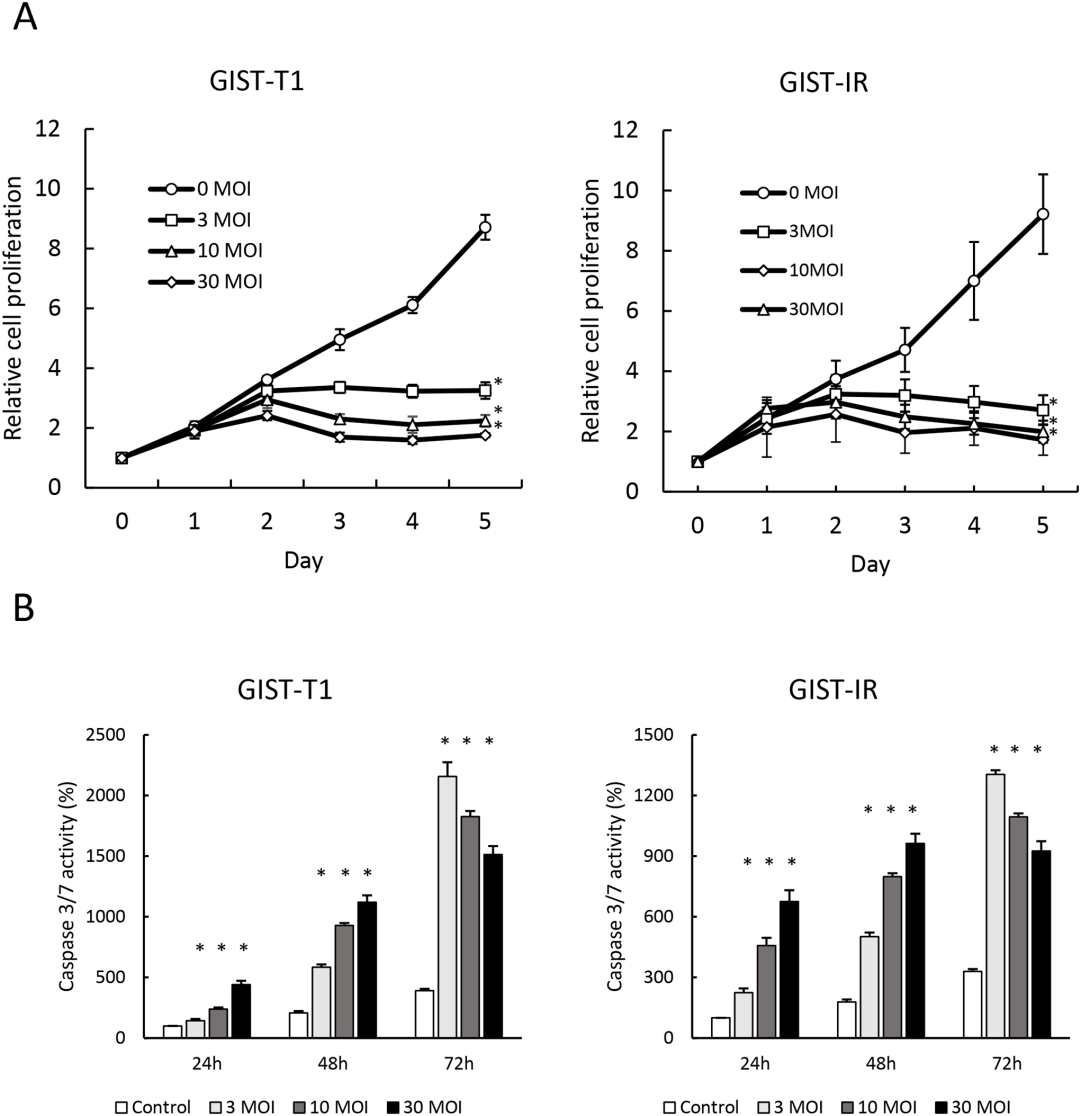
Growth inhibition and apoptosis induction by reovirus in GIST-T1 and GIST-IR cells *in vitro* **(A)** 3.0 × 10^3^ GIST-T1 or GIST-IR cells were plated in 96-well plates and treated with reovirus, and cell proliferation was measured by WST-8 assay. **(B)** 5.0 × 10^3^ GIST-T1 or GIST-IR cells were plated in 96-well plates and treated with 3, 10, and 30 multiplicity of infection (MOI) of reovirus. Apoptosis induction by reovirus in each cell type was evaluated by measuring caspase-3/7 activity. Each result represents the mean ± SD of three experiments performed in triplicate. ^*^P<0.01, compared to controls.

**Figure 3:**
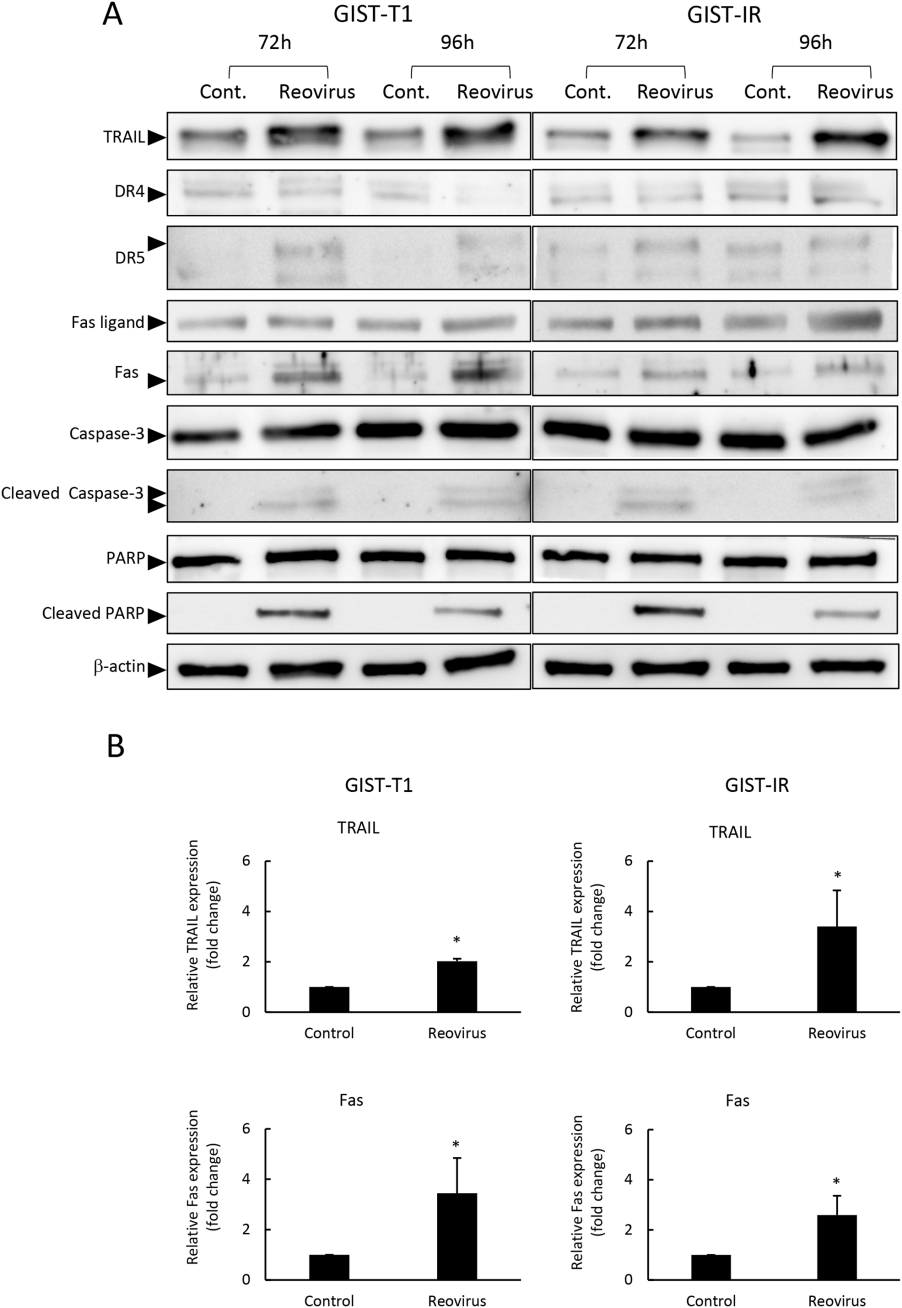
Reovirus enhanced TRAIL and Fas expression in GIST-T1 and GIST-IR cells **(A)** Western blot analysis showed changes in apoptosis-related proteins, TRAIL, cell surface death receptors (DR) 4, DR5, Fas ligand, Fas, caspase-3, and poly ADP ribose polymerase (PARP) in GIST-T1 and GIST-IR cells following treatment with 10 MOI of reovirus for 72 or 96 hours. **(B)** Relative expression of TRAIL and FAS proteins in GIST-T1 and GIST-IR with or without 10 MOI of reovirus for 96 hours quantified by densitometry using imageJ software. Each result represents the mean ± SD of three experiments. ^*^P<0.01, compared to controls.

We evaluated the cytotoxicity of reovirus against GIST-T1 and GIST-IR cells. After treatment with reovirus for five days, cell proliferation decreased significantly in both GIST-T1 and GIST-IR cells in a virus concentration-dependent manner (Figure 2A). Caspase 3/7 activities of cells treated with reovirus were higher than those of the control at all time points (p < 0.01) (Figure 2B). Moreover, caspase inhibitors including BID inhibitor and caspase 3/7 inhibitor significantly suppressed the caspase 3/7 activities induced by reovirus treatment in both GIST-T1 and GIST-IR (Supplementary Figure 3). In accordance with the results of the caspase assays, Western blots showed that reovirus induced both activation of caspase-3 and PARP cleavage in GIST-T1 and GIST-IR cells after reovirus treatment (Figure 3).

### Molecular response after reovirus treatment in GIST cells

To understand the molecular mechanisms responsible for apoptosis induced by reovirus in GIST cells, we measured gene expression in GIST-T1 cells treated with reovirus, using a human polymerase chain reaction (PCR) array consisting of 84 apoptosis-related genes. In GIST-T1 cells with reovirus treatment, several apoptosis-related genes showed higher mRNA levels than those in GIST-T1 cells without reovirus treatment (Table 2). Among these candidate genes that account for apoptosis caused by reovirus in GIST-T1 cells, we focused on two apoptosis-related genes, TNF-related apoptosis-inducing ligand (TRAIL) and Fas. TRAIL is known as one of the key factors promoting apoptosis induced by reovirus [22, 23], and we previously reported that the apoptosis-signaling pathway stimulated by TRAIL plays a crucial role in the cytotoxic effect of reovirus against gastric cancer [22]. Furthermore, we investigated the role of Fas in reovirus-induced cell death. Rikhof et al. reported the efficacy of Fas ligand (FasL) against GIST was independent of imatinib sensitivity, and proposed the utility of Fas, which is expressed abundantly in GIST samples, as a potential therapeutic target for GIST [24]. Western blotting analysis demonstrated that TRAIL and Fas were expressed in GIST-T1 and GIST-IR cells, and their expression levels were increased after reovirus treatment (Figure 3A, 3B). Fas protein expression levels were significantly increased, whereas no significant changes were seen in DR4 and DR5 expression after reovirus treatment in either GIST-T1 or GIST-IR cells (Figure 3A).

**Table 2:** Expression profiles of apoptosis-related genes displaying at least 10-fold change in expression after treatment with 10 MOI of reovirus in GIST-T1 cells

symbol	GenbnkID	Increase	Gene name
BCL2A1	NM_00409	112.53	BCL2-related protein A1
BIK	NM_001197	17.31	BCL2-interacting killer
CASP1	NM_033292	110.19	Caspase 1, apoptosis-related cysteine peptidase
CASP10	NM_001230	14.56	Caspase 10, apoptosis-related cysteine peptidase
CASP4	NM_001225	18.61	Caspase 4, apoptosis-related cysteine peptidase
CD40	NM_001250	21.03	CD40 molecule, TNF receptor superfamily member 5
CD70	NM_001252	741.88	CD70 molecule
FAS	NM_000043	14.66	Fas (TNF receptor superfamily, member 6)
GADD45A	NM_001924	28.11	Growth arrest and DNA-damage-inducible, alpha
HRK	NM_003806	255.63	Harakiri, BCL2 interacting protein
LTA	NM_000595	44.36	Lymphotoxin alpha
RIPK2	NM_003821	45.83	Receptor-interacting serine-threonine kinase 2
TNF	NM_000594	776.28	Tumor necrosis factor
TNFRSF10B	NM_003842	18.78	Tumor necrosis factor receptor superfamily, member 10b
TNFRSF9	NM_001561	887.03	Tumor necrosis factor ligand superfamily, member 9
TNFSF10	NM_003810	15.02	Tumor necrosis factor ligand superfamily, member 10

### Role of Fas-FasL pathway in reovirus-induced apoptosis in GIST cells

**Figure 4:**
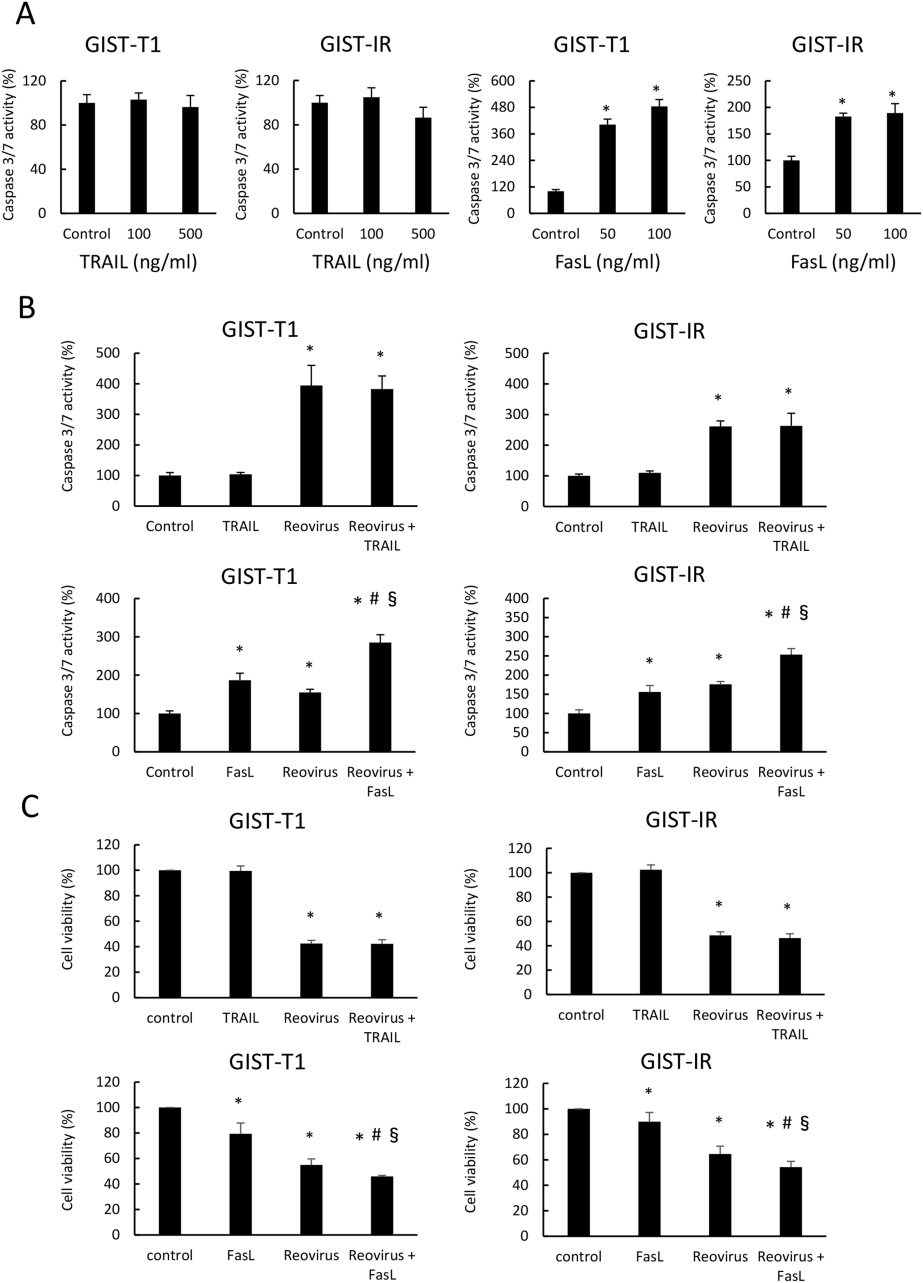
Combination treatment of FasL and reovirus significantly induced apoptosis in GIST-T1 and GIST-IR cells **(A)** Caspase 3/7 activity was measured in GIST-T1 and GIST-IR cells treated with TRAIL or FasL for 24 hours. **(B)** Caspase 3/7 activity was evaluated in GIST-T1 and GIST-IR cells treated with 10 MOI reovirus treatments for 24 hours, and subsequently treated with TRAIL or FasL for 24 hours. **(C)** Cell viability was evaluated in GIST-T1 and GIST-IR cells treated with 10 MOI reovirus treatments for 48 hours, and subsequently treated with TRAIL or FasL for 48 hours. Each result represents the mean ± SD of three experiments performed in triplicate. ^*^P <0.01, compared to control; ^#^P <0.01, compared to FasL alone; ^§^P <0.01, compared to reovirus alone.

To investigate whether TRAIL and FasL are implicated in reovirus-induced apoptosis, we evaluated caspase 3/7 activity and cell viability in GIST-T1 and GIST-IR cells after treatment with TRAIL alone, FasL alone, TRAIL in combination with reovirus, or FasL in combination with reovirus. TRAIL alone did not cause any damage to either GIST-T1 or GIST-IR cells, whereas TRAIL alone induced apoptosis in MKN28, a gastric cancer cell line that expresses abundant DR5 (data not shown here). In contrast, FasL significantly induced apoptosis and suppressed cell viability in both GIST-T1 and GIST-IR cells (Figure 4A). Furthermore, the combination treatment with reovirus and FasL enhanced caspase 3/7 activities and showed the ability to inhibit cell growth in both GIST-T1 and GIST-IR cells. On the other hand, TRAIL did not enhance caspase 3/7 activities in GIST-T1 or GIST-IR cells treated with reovirus (Figure 4B, 4C).

### Effect of reovirus on growth of GIST-T1 and GIST-IR cells *in vivo*

**Figure 5:**
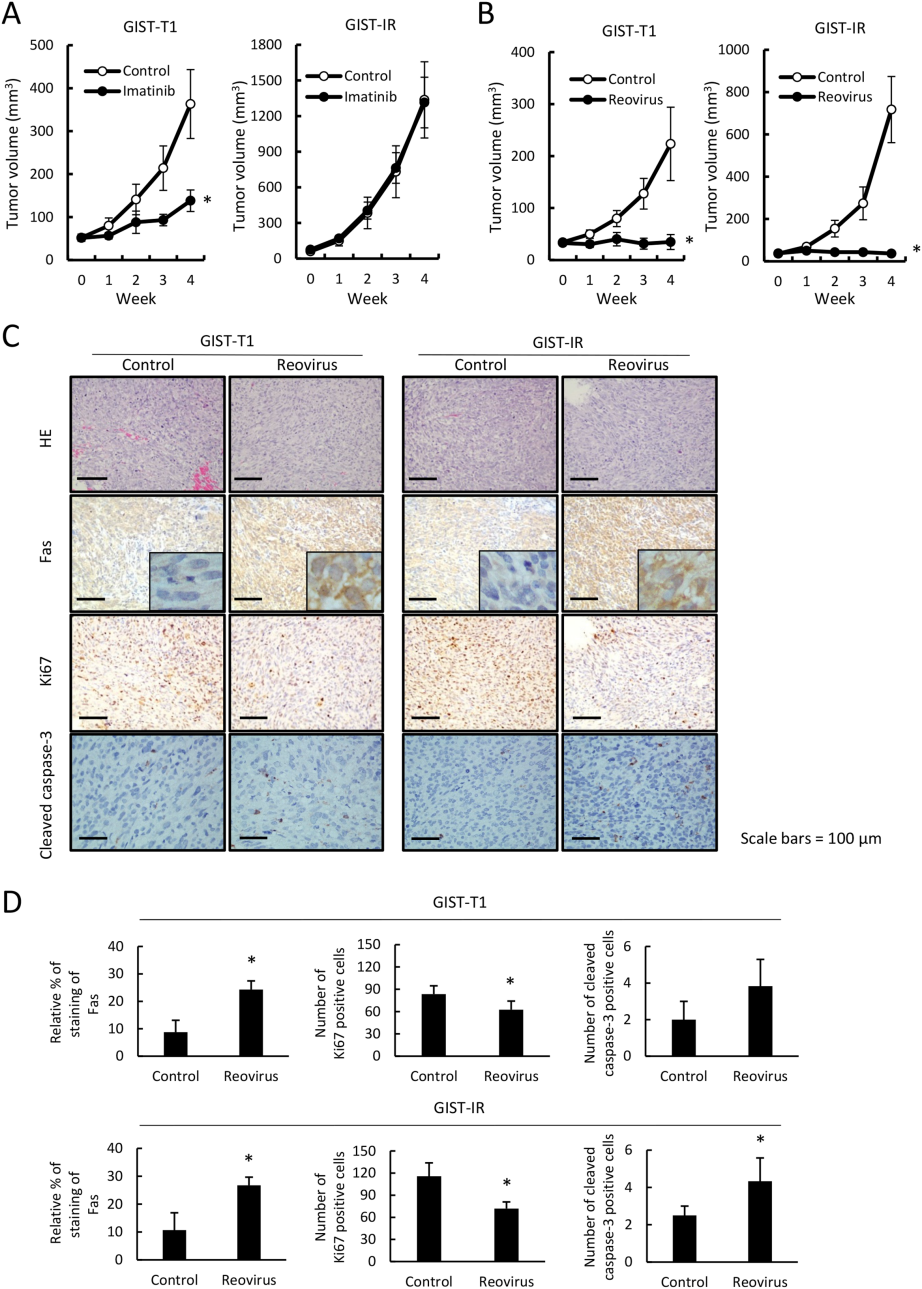
Imatinib resistance in GIST-IR cells and efficacy of reovirus therapy against GIST-T1 and GIST-IR cells *in vivo* Groups of BALB/c nude mice (n=5 mice per group) were injected subcutaneously with 3 × 10^6^ cells of GIST-T1 or GIST-IR cells, and grown to palpable size for 14 days. **(A)** Mice were treated with imatinib dissolved in water at a dose of 40 mg/kg/day or water alone by oral administration for 4 weeks. **(B)** Mice were treated with a direct intra-tumor injection of 1×10^8^ pfu reovirus weekly for 4 weeks. Tumor volumes were estimated once a week. Data are shown as means ± SE ^*^P < 0.05, compared to control. **(C)** Representative images of hematoxylin and eosin (HE) and Fas, Ki67, and Cleaved caspase-3 staining of subcutaneously transplanted GIST-T1 and GIST-IR cells treated with or without injection of 1×10^8^ pfu reovirus weekly for 4 weeks. **(D)** Quantification of Fas expression, Ki67-positive cells and cleaved caspase-3-positive cells in GIST-T1 or GIST-IR xenografts with or without injection of 1×10^8^ pfu reovirus weekly for 4 weeks. Data are shown as means ± SD ^*^P < 0.05, compared to controls.

We examined whether reovirus treatment could efficiently inhibit tumor growth of GIST-T1 and GIST-IR cells using a xenograft mouse model *in vivo*. First, we confirmed that tumors derived from GIST-IR cells displayed resistance to imatinib, whereas GIST-T1-derived tumors showed sensitivity to imatinib treatment (Figure 5A). Next, we investigated whether reovirus treatment shows antitumor activity against GIST-T1 or GIST-IR cells subcutaneously transplanted in nude mice. Reovirus treatment significantly inhibited tumor growth in GIST-T1 xenografts and also in GIST-IR xenografts without adverse effects (Figure 5B). Histopathological analyses of extracted tumors revealed transplanted GIST-T1 and GIST-IR tumors exhibited more apoptotic cells after reovirus therapy compared with the control. Moreover, Fas protein expression was significantly higher in GIST-T1 and GIST-IR tumor xenografts in mice treated with reovirus relative to those in mice without reovirus treatments (Figure 5C, 5D). In addition, the number of Ki67-positive cells was significantly decreased in GIST-T1 and GIST-IR cells treated with reovirus, and the number of cleaved caspase-3-positive cells was increased in GIST-T1 and GIST-IR cells (Figure 5D).

## DISCUSSION

Although the antitumor effects of reovirus against various types of carcinoma have been reported, the efficacy of reovirus against GIST cells required clarification. In this study, we demonstrated that reovirus has a powerful oncolytic effect on GIST and imatinib-resistant GIST cells, respectively.

Currently, imatinib is the first-line standard therapy for unresectable, metastatic, or recurrent GIST [25–28]. The activity and efficacy of imatinib in advanced GIST patients has been reported, showing that GISTs with specific mutations (such as PDGFRA exon18, D842V mutation, or KIT exon17, D816V mutation) have primary resistance to imatinib [29]. In addition, the development of secondary resistance to imatinib prevents highly effective therapy against GIST. Multitargeted RTK inhibitors, sunitinib or regorafenib, are often applied for the treatment of imatinib-resistant GIST; however, they have limited efficacy and demonstrate higher rates of adverse events as compared to imatinib [4, 7, 30]. Therefore, the development of novel therapeutic options is crucial to overcoming these obstacles in conventional therapy for GIST patients.

Reovirus infection induces few minor symptoms including cough, sore throat, and diarrhea. Even though reovirus infections are mild and sometimes subclinical, reovirus displays selective oncolytic activity against transformed and malignant cells [9, 18, 31, 32]. Previous studies on the mechanisms of reovirus-induced killing of tumor cells suggested that Ras pathway activation was a key determinant of viral replication and subsequent oncolysis [33, 34]. Approximately 90% of GISTs gained functional mutations in either *KIT* or *PDGFRA* that consistently drive RTK pathways without activation by their ligands. Activated RTKs subsequently lead to activation of the Ras pathway, and thus we speculated that reovirus might display an antitumor effect against GIST. Indeed, our present study revealed that reovirus treatment induced apoptotic cell death in GIST cells regardless of their sensitivity to imatinib (Figures 2A, 4B). However, it remains unclear whether reovirus has antitumor activity against primary imatinib-resistant GIST cells. In addition, the efficacy of reovirus against secondary imatinib-resistant GIST with acquired point mutations to *KIT* in exon 13, 14, and 17, which is detected in 40-80% of patients progressing on imatinib, and *PDGFRA* exon 18 mutation, identified in a subset of GIST refractory to imatinib [2, 19, 20, 35], is not clarified in this study. We could not identify any of these specific mutations related to secondary imatinib-resistance in GIST-IR cells. Another possible mechanism of imatinib resistance is *KIT* gene amplification, and other oncogenes and tumor suppressor genes may also be responsible for imatinib resistance in GIST, for example, silencing of the *PTEN* gene or activation of ERK [2, 36, 37]. Furthermore, it is reported that PI3K/Akt/mammalian target of rapamycin (mTOR) signaling appears particularly important in imatinib-resistant GIST [35, 38, 39]. In our established GIST-IR cells, RTK phosphorylation arrays revealed the activation of tyrosine kinase receptors and their downstream targets, despite treatment with high concentrations of imatinib (Figure 1C, 1D, Supplementary Figure 1), which enable GIST-IR cells to resist imatinib cytotoxicity.

As mentioned above, we focused on TRAIL and Fas as two key molecules among the genes involved in reovirus-induced apoptosis. TRAIL belongs to the tumor necrosis factor (TNF) family of proteins and induces apoptosis through its receptors DR4 and DR5 in a wide variety of tumor cells, but does not cause toxicity in the majority of normal cells [40]. We have previously reported that reovirus-induced apoptosis is mediated by TRAIL and is associated with its release from infected cells in gastric cancer cells. Others have also reported that reovirus promotes apoptosis through the expression of DR5 and the release of TRAIL from reovirus infected cells, and exogenous TRAIL was reported to enhance reovirus-induced apoptosis [22, 23, 41]. In the present study, TRAIL did not induce apoptosis in GIST cells, and TRAIL did not enhance the cytotoxic effects of reovirus on GIST cells even after reovirus stimulation. In GIST cells, TRAIL expression is enhanced as a result of reovirus-induced apoptosis, even though TRAIL may not be involved in the mechanism of reovirus action. In contrast, Fas also belongs to the TNF family of death receptors, and induces apoptosis when bound to its ligand, FasL. Studies have demonstrated that some cancer cells are susceptible to Fas agonistic antibodies [42, 43]. As for the correlation between reovirus and Fas, it has been reported that Fas is implicated in reovirus-mediated apoptotic signaling in the mouse brain [44]. We demonstrated that FasL mediated apoptosis in GIST-T1 and GIST-IR cells (Figure 4A), in accordance with previous reports [24]. Moreover, we demonstrated the combination effects of reovirus and FasL against GIST-T1 and GIST-IR cells (Figure 4B). These results indicate that the Fas-FasL pathway plays an important role in reovirus-induced apoptosis in GIST cells regardless of their resistance to imatinib. Although the current study focused on the antitumor activity of reovirus for GIST as monotherapy, our study suggests the potential utility of combination treatment of reovirus and FasL against GIST.

The present *in vivo* study demonstrated that four-week treatment with imatinib showed a modest antitumor effect against GIST-T1 cells. Subcutaneous transplanted GIST-IR cells were completely resistant to imatinib treatment in our xenograft model. In contrast, significantly slower tumor growth was observed in mice treated with reovirus without side effects, such as weight loss and diarrhea, in both GIST-T1 and GIST-IR cells (Figure 5B, Supplementary Figure 4). Histopathologically, reovirus induced apoptotic changes and reduced cell growth activity in xenograft tumor cells. Reovirus also enhanced Fas expression in transplanted GIST-T1 and GIST-IR cells, which also indicated the importance of the Fas-FasL pathway in reovirus-induced apoptosis.

In conclusion, we demonstrated that oncolytic reovirus induced apoptosis and suppressed tumor growth in GIST, regardless of its sensitivity to imatinib. Despite the limitations of our imatinib-resistant GIST model, reovirus might have potential utility as an alternative GIST therapy, irrespective of imatinib sensitivity. This is because the mechanism of reovirus antitumor activity is completely different from RTK inhibitors such as imatinib. Furthermore, primary and secondary imatinib resistance is mainly attributable to mutations of RTKs that interfere with drug binding. Moreover, reovirus therapy is potentially more advantageous than the conventional GIST therapy with its associated adverse effects.

## MATERIALS AND METHODS

### Reagents

Imatinib was purchased from Sigma-Aldrich (St. Louis, MO) and dissolved in DMSO to a stock concentration of 10^−2^ M, and then stored at −80°C until use. The DMSO concentration of the medium did not exceed 0.01% in order to avoid any effect on GIST-T1 cell viability.

### Cell culture

The human GIST cell line, GIST-T1, provided by COSMO BIO (Tokyo, Japan), was established and has been characterized in detail by Taguchi et al. [45]. GIST-T1 has an activating mutation in *KIT* exon 11 and is sensitive to imatinib. The cells were originally purchased in 2014, and all experiments in this study were carried out using cells within 20 passages. Cells were authenticated through morphological examination and expression of c-kit. Imatinib-resistant GIST, GIST-IR, was established by culturing cells with increasing concentrations of imatinib (5-80 nM) for 6 months [36, 37]. GIST-T1 and GIST-IR were cultured in high (4500 mg/L) glucose Dulbecco’s Modified Eagle’s Medium (Wako Pure Chemical Industries, Osaka, Japan) supplemented with 10% FBS under 5% CO_2_ at 37°C. Microbial contamination test of both cell lines was conducted by an outsourced organization, ICLAS Monitoring Center (Kanagawa, Japan).

### Mutation analysis of KIT and PDGFRA

Sequence analysis was utilized to examine whether secondary mutations occurred in *KIT* and *PDGFRA* genes in GIST-IR cells. Total RNA was isolated with 16 LEV simplyRNA Cells and Tissue Kits (Promega, Madison, WI) and the same amounts of RNA were reverse-transcribed into cDNA using a High-Capacity cDNA Reverse Transcription kit (Applied Biosystems, Tokyo, Japan) according to the manufacturer’s instructions. Primer sets for PCR are shown in Table 1. PCR was carried out using a TaKaRa PCR Thermal Cycler (Takara, Shiga, Japan), and purified PCR products were sequenced by the ABI PRISM 310 Genetic Analyzer (Applied Biosystems, Foster City, CA). The amplification protocol was 94°C for 1 minute, then 30 cycles at 98°C for 10 seconds, 68°C for 30 seconds.

### Cell viability assay

Cell viability was analyzed using the WST-8 cell proliferation assay. Cells were seeded into 96-well culture plates at a concentration of 5 × 10^3^ cells/100 μL/well and incubated overnight. Cells were then exposed to imatinib within the concentration range of 25 - 3200 nM on day 0. Cell survival was evaluated using the Cell Counting Kit-8 (Dojindo Laboratories, Kumamoto, Japan) according to the manufacturer’s protocol, and the absorption at 450 nm was measured with a microplate spectrophotometer (SPECTRA MAX340; Molecular Devices). Furthermore, to test the combination efficacy of reovirus with TRAIL or FasL, GIST-T1 and GIST-IR cells were infected with reovirus at 10 MOI, and after 48 hours, cells were treated with TRAIL or FasL. Forty-eight hours after TRAIL or FasL treatment, cell viability was assessed using the Cell Counting Kit-8. All experiments were performed in more than five samples and repeated at least three times. Viability inhibition (%) was calculated as follows: (measured value of treated group - blank) / (measured value of control group - blank) × 100%. The IC50 was calculated by probit analysis.

### Cell proliferation assay

Cell proliferation was analyzed using the WST-8 cell proliferation assay. Cells were seeded into 96-well culture plates at a concentration of 3 × 10^3^ cells/100 μL/well and incubated overnight. Cells were then treated with reovirus at multiplicity of infection (MOI) of 3, 10, and 30 on day 0. Cell proliferation was evaluated using the Cell Counting Kit-8 (Dojindo Laboratories, Kumamoto, Japan) according to the manufacturer’s protocol, and the absorption at 450 nm was measured with a microplate spectrophotometer (SPECTRA MAX340; Molecular Devices). We observed proliferative capacity, calculating the values from day 1 to day 5 with the number of cells on day 0 set to 1.

### Apoptosis assays

Apoptosis induced by reovirus treatment was quantified by measuring caspase-3/7 activity with the Caspase-Glo 3/7 Assay System (Promega). GIST-T1 and GIST-IR cells were seeded into 96-well culture plates at a concentration of 3 × 10^3^ cells/100 μL/well and incubated overnight, and then treated with reovirus (10 MOI), tumor necrosis factor-related apoptosis-inducing ligand (TRAIL) or Fas ligand (FasL). After the indicated times, the caspase-3/7 activity of the cells was measured by adding an aliquot of the homogenous caspase-3/7 reagent to each well, incubating the plate for 1 hour at room temperature, and measuring the fluorescence intensity with a fluorescent plate reader. Furthermore, caspase-3/7 activities of the cells treated with 10 MOI reovirus in combination with caspase 3/7 inhibitor (SANTA CRUZ Biotechnology) or BID inhibitor (SANTA CRUZ Biotechnology) were measured using the Caspase-Glo 3/7 Assay System. To test the combination efficacy of reovirus with TRAIL or FasL, GIST-T1 and GIST-IR cells were treated with TRAIL or FasL, and after 24 hours, cells were infected with reovirus at 10 MOI. Forty-eight hours after reovirus treatment, cell apoptosis was assessed using the Caspase-Glo 3/7 Assay System.

### Quantitative reverse transcription–polymerase chain reaction

To investigate differences in gene expression induced by reovirus infection, reverse transcription–polymerase chain reaction (RT-PCR) was performed using a human apoptosis RT^2^ Profiler PCR array (Qiagen, Hilden, Germany) containing 84 different apoptosis-associated genes and five housekeeping genes (ACTB, B2M, GAPDH, HPRT1, and RPLP0). We assessed the mRNA expression profiles of GIST-T1 and GIST-IR cells treated with reovirus at 10 MOI for 48 hours. Real-time quantitative RT-PCR analyses were performed using an ABI 7500 Fast Real-Time PCR system (Applied Biosystems) according to the manufacturer’s recommendations. Results were analyzed using PCR Array Data Analysis on the Qiagen web page (http://www.qiagen.com/us/shop/genes-and-pathways/data-analysis-center-overview-page/). To normalize transcript levels, we chose the “automatic selection from full plate” method.

### Western blotting

Cells were washed with PBS (Sigma-Aldrich) 3 times and then added to 1 mL of cell lysis buffer (Cell Signaling Technology, Danvers, MA) containing 20 mmol/L Tris-HCl (pH 7.5), 150 mmol/L NaCl, 1 mmol/L Na_2_EDTA, 1 mmol/L EGTA, 1% Triton, 2.5 mmol/L sodium pyrophosphate, 1 mmol/L β-glycerophosphate, 1 mmol/L Na_3_VO_4_, and 1 μg/mL leupeptin. Cells were disrupted for 15 seconds on ice using a Bioruptor sonicator (Cosmo Bio, Tokyo, Japan) and centrifuged at 15,000 rpm for 20 minutes at 4°C. Each sample was normalized to an equal protein concentration using a Protein Assay Kit (Bio-Rad Laboratories, Hercules, CA). Equal quantities of 2× SDS-PAGE sample buffer [0.5 M Tris-HCl (pH 7.2), 1% SDS, 100 mmol/L β-mercaptoethanol, and 0.01% bromophenol blue] were added to each sample, and samples were boiled for 5 minutes at 100°C. Aliquots of each sample were separated by SDS-PAGE on a 10% gel and transferred to a nitrocellulose membrane. The membrane was blocked with 5% skim milk in PBS for 1 hour at room temperature, followed by incubation with the primary antibodies against TRAIL, cell surface death receptor (DR) 4, DR5, Fas, FasL (Abcam, Cambridge, MA), phosphorylated c-Kit (Tyr719), phosphorylated Akt (Ser473), phosphorylated ERK1/2 (Thr202/Tyr204), caspase-3, cleaved caspase-3, poly ADP ribose polymerase (PARP), cleaved PARP (Cell Signaling Technology) and β-actin (Sigma-Aldrich) overnight at 4°C. Membranes were washed with 0.05% Tween 20 in PBS 3 times at 5-minutes intervals, incubated with the secondary antibody for 1 hour at room temperature, and washed again with 0.05% Tween 20 in PBS 3 times at 5-minute intervals. The membrane was incubated with Western blotting detection reagent, ECL Prime (GE Healthcare, Buckinghamshire, UK) for 5 minutes at room temperature and filmed using ImageQuant LAS 4000 (GE Healthcare). Quantification of Fas and TRAIL protein expression corrected with β-actin in western blotting was performed using ImageJ image analysis software (https://imagej.nih.gov/ij).

### Receptor tyrosine kinase phosphorylation array

PathScan^®^ RTK Signaling Antibody Array Kit (Cell Signaling Technology) is a slide-based antibody array founded upon the sandwich immunoassay principle. The array kit allows for the simultaneous detection of 28 receptor tyrosine kinases and 11 important signaling nodes, when phosphorylated at tyrosine or other residues. Target-specific capture antibodies have been spotted in duplicate onto nitrocellulose-coated glass slides. Whole cell lysates of GIST-T1 and GIST-IR cells exposed to imatinib are incubated on the slide, followed by a biotinylated detection antibody cocktail. Streptavidin-conjugated HRP and LumiGLO® reagent were then used to visualize the bound detection antibody by chemiluminescence. Slides were imaged using ImageQuant LAS 4000 (GE Healthcare). Quantification of tyrosine kinase receptors and cell signaling intermediates in the receptor tyrosine kinase (RTK) phosphorylation array was performed using ImageJ image analysis software.

### Tumor xenograft experiments

Pathogen-free female nude mice (BALB/c Slc-nu/nu), 6 to 8 weeks of age, with a body weight of 18 to 22 g, were obtained from Shizuoka Laboratory Animal Center (Hamamatsu, Japan). Xenograft tumor models were established by subcutaneously implanting 3 × 10^6^ GIST-T1 or GIST-IR cells in 100 μL of PBS and equivalent amounts of matrigel (Corning, NY). Mice were then treated with imatinib or reovirus. Imatinib was dissolved in a small amount of DMSO and added to water. Some of the mice were treated daily with 40 mg/kg imatinib by oral administration for 4 weeks (n=5). The other mice were treated with direct intratumor injections of reovirus at a dose of 1 × 10^8^ plaque-forming units (pfu) per mouse weekly for 4 weeks (n=5). The dose of reovirus was chosen based on our previous study [22]. The maximum tumor diameter (L) and the diameter at right angles to that axis (W) were measured weekly using calipers, and the volume was calculated according to the formula, (L × W^2^)/2 [22, 37]. Tumor size was measured and anti-tumor activity of imatinib or reovirus was evaluated until 4 weeks after treatment. Subsequently, transplanted tumors were excised and fixed in formalin for histological analysis after euthanasia. There were no adverse effects, including weight loss and infection, observed throughout the treatment period. The procedures in these experiments were approved by the Nagoya City University Center for Experimental Animal Science, and the mice were cared for according to the guidelines of the Nagoya City University for Animal Experiments.

### Immunohistochemistry

Subcutaneous tumors in nude mice were removed and fixed in 10% buffered formalin for 24 hours. Formalin-fixed and paraffin-embedded sections (4 μm) were used for immunohistochemistry. Sections were stored dry until use for histological analysis. After pre-incubation with 0.3% hydrogen peroxide in methanol for 30 minutes to block endogenous peroxidase activity, sections were rinsed in PBS. Fas, Ki67 and cleaved caspase-3 were visualized immunohistochemically by the streptavidin-biotin method using 1:400 rabbit anti-Fas antibody (Abcam), 1:100 rabbit anti-Ki67 antibody (Leica Biosystems, Nussloch, Germany), or 1:400 rabbit anti-cleaved caspase-3 antibody (Cell Signaling Technology) and the Histofine SAB-PO® Kit (Nichirei, Tokyo, Japan), according to the manufacturer’s protocol. Sections were rinsed and incubated sequentially with secondary antibody (goat biotinylated anti-rabbit IgG antibody) and with the streptavidin-biotin-peroxidase complex. Sections were then washed with PBS, incubated in diaminobenzidine solution containing 0.003% hydrogen peroxide and 10 mM sodium azide, followed by a Mayer hematoxylin counterstain, and the peroxidase reaction was developed. Sections were then dehydrated through graded ethanol solutions (70%-100%). For quantification, the number of Ki67-positive cells or cleaved caspase-3-positive cells in the field, approximately 70,000 μm^2^, was counted at 400× magnification randomly. Only cells stained precisely were counted as Ki67-positive or caspase-3-positive cells to avoid counting cells with non-specific staining. For objective cell counts, two independent researchers identified the positive-stained cells blindly; then observers, blinded to the treatment condition, performed the cell counts. The counts from six fields were averaged. Observations were obtained from at least three independent experiments. To quantify Fas protein expression in the sections at 200× magnification, we used ImageJ software with immunohistochemistry profiler plugin as described previously by Varghese et al [46].

### Statistical analysis

All statistical analyses were performed with EZR (Saitama Medical Center, Jichi Medical University, Saitama, Japan), which is a graphical user interface for R (The R Foundation for Statistical Computing, Vienna, Austria). More precisely, it is a modified version of R commander designed to add statistical functions frequently used in biostatistics [47]. Mann-Whitney U test was used for continuous variables. P values of less than 0.05 were considered to indicate statistical significance.

## SUPPLEMENTARY MATERIALS FIGURES AND TABLES



## Abbreviations

GIST: Gastrointestinal stromal tumor
PDGFRA: Platelet-derived growth factor receptor alpha
RTK: Receptor tyrosine kinase
PCR: Polymerase chain reaction
TRAIL: TNF-related apoptosis-inducing ligand
FasL: Fas ligand
TNF: Tumor necrosis factor
