# Supplementary material for: Anti-tumor efficacy of oncolytic reovirus against gastrointestinal stromal tumor cells
Source: Oncotarget. 2017 Dec 18;8(70):115632–46. doi: 10.18632/oncotarget.23361 (PMC5777799; doi:10.18632/oncotarget.23361)
Supplement: Supplementary file 1 [file oncotarget-08-115632-s001.pdf]

Anti-tumor efficacy of oncolytic reovirus against gastrointestinal stromal tumor cells

SUPPLEMENTARY MATERIALS

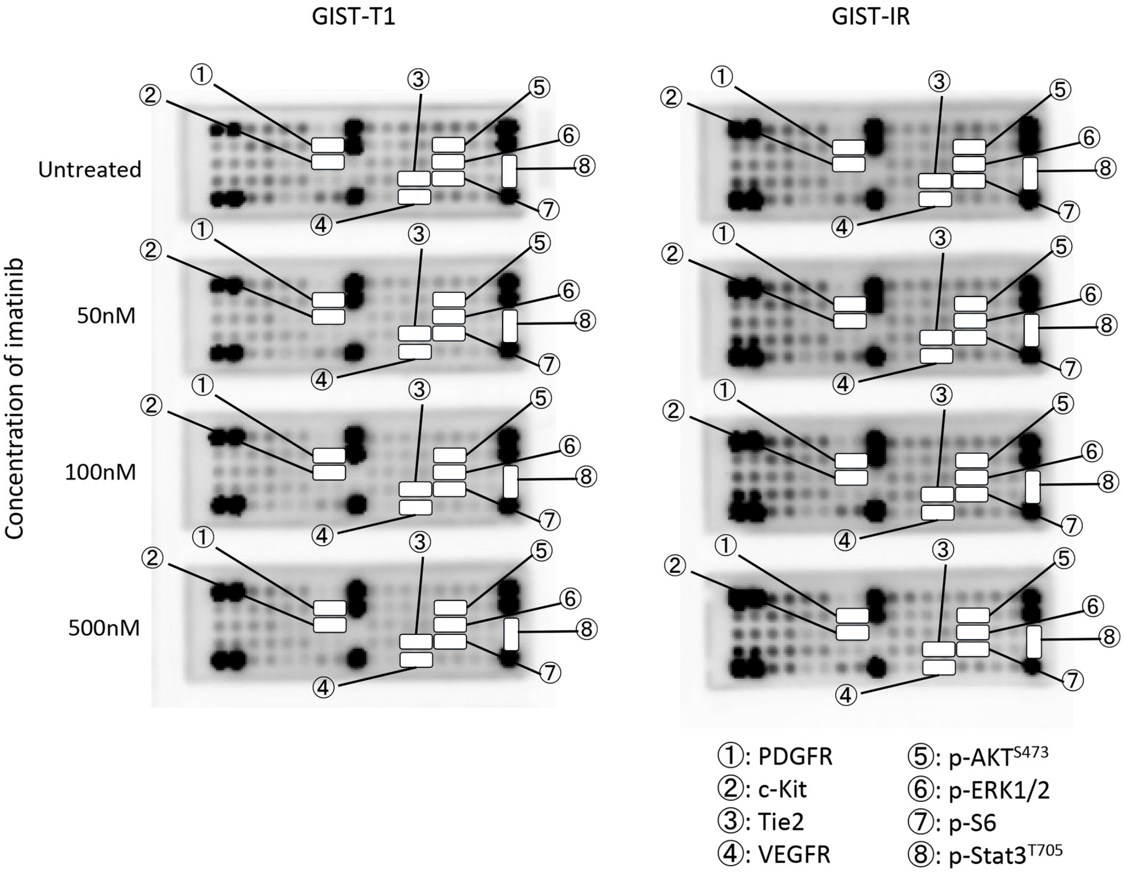

Supplementary Figure 1: Chemiluminescent array images captured by ImageQuant LAS 4000. Each dot represents the expression of activated tyrosine kinase receptors and downstream signals in GIST-T1 and GIST-IR cells treated with the indicated concentration of imatinib.

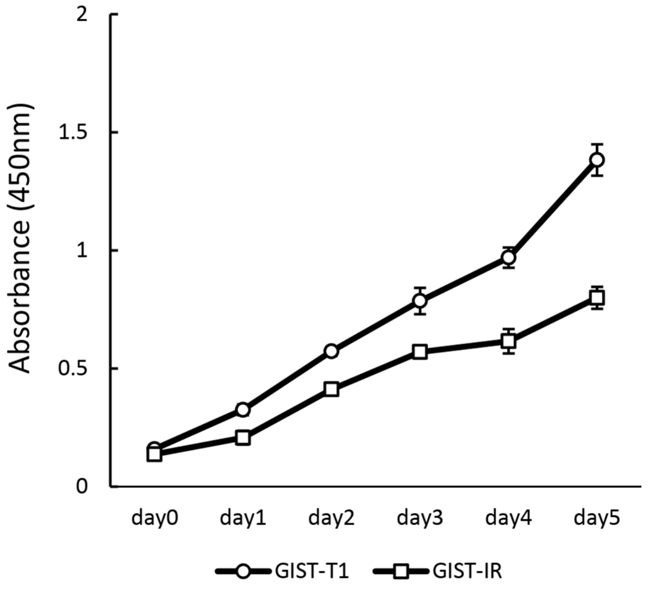

Supplementary Figure 2: 3.0 × 10^3 GIST-T1 or GIST-IR cells were plated in 96-well plates, and cell proliferation was measured using the Cell Counting Kit-8. The absorbance of each sample was determined using a microplate reader at 450 nM.

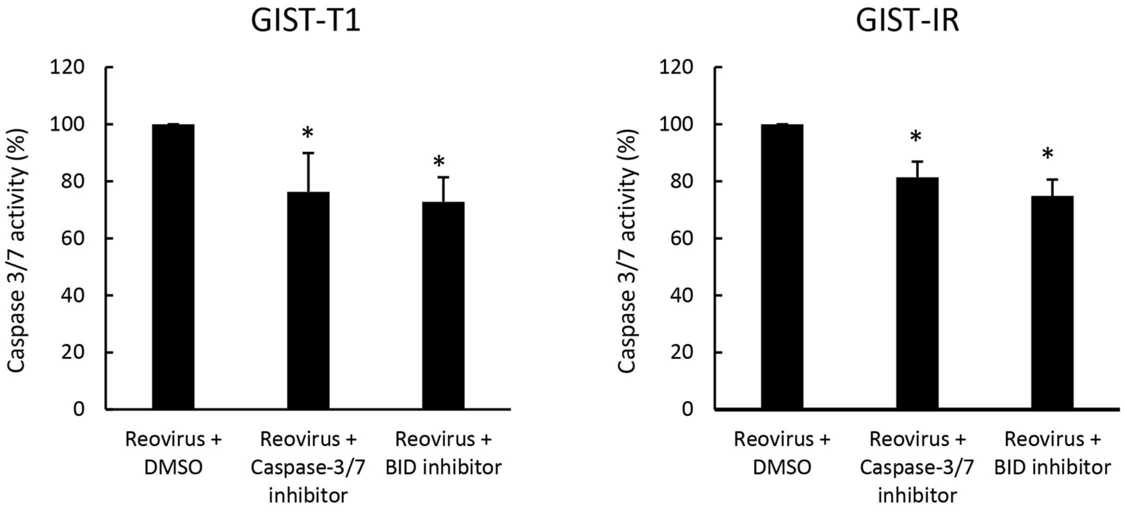

Supplementary Figure 3: Caspase 3/7 activity was measured in GIST-T1 and GIST-IR cells treated with 10 MOI reovirus treatments for 24 hours, and subsequently treated with 50 µM caspase 3/7 inhibitor or 50 µM BID inhibitor for 24 hours. Each result represents the mean ± SD of three experiments performed in triplicate. *P <0.01, compared to reovirus alone.

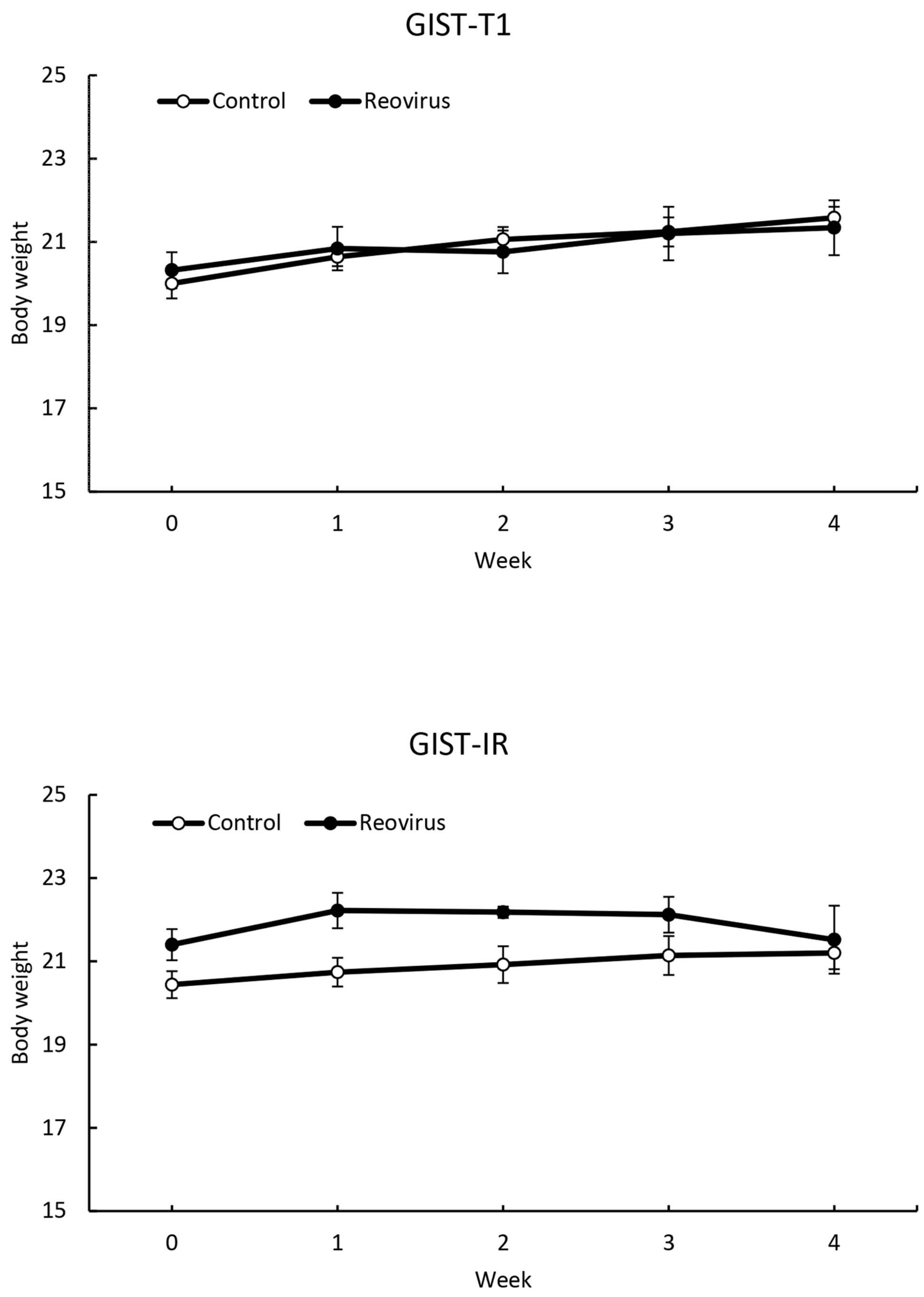

Supplementary Figure 4: BALB/c nude mice were injected subcutaneously with 3.0 × 10^6 cells of GIST-T1 or GIST-IR cells, and subsequently treated with or without 1.0 × 10^8 pfu reovirus weekly for 4 weeks. The weights of the mice were measured weekly through the experiment. N=5 mice per group. Data are shown as means ± SD.
